# Massive enteric necrosis caused by histiocytic sarcoma embolism: a case report

**DOI:** 10.1186/s40792-024-02031-4

**Published:** 2024-10-04

**Authors:** Yoshitaka Imoto, Masato Yamadera, Hiroki Ohno, Koichi Okamoto, Yoshiki Kajiwara, Yoji Kishi, Hideyuki Shimazaki, Susumu Matsukuma, Hideki Ueno

**Affiliations:** 1 Department of Surgery, National Defense Medical College, Namiki 3-2, Tokorozawa, Saitama 359-8513 Japan; 2 Department of Laboratory Medicine, National Defense Medical College Hospital, National Defense Medical College, Tokorozawa, Saitama 359-8513 Japan; 3 Department of Pathology and Laboratory Medicine, National Defense Medical College, Tokorozawa, Saitama 359-8513 Japan

**Keywords:** Histiocytic sarcoma, Enteric necrosis, Tumor embolism

## Abstract

**Background:**

Histiocytic sarcoma (HS) is a rare disease characterized by the presence of neoplastic histiocytes. We herein report an unusual case of HS that caused massive tumor embolism-related transmural necrosis of the small intestine.

**Case presentation:**

A 64-year-old man presented with multiple nodules in the lungs, bone, mediastinum, and subcutaneous tissues that were incidentally detected on preoperative computed tomography for early transverse colon cancer. Approximately two months later, the patient presented with signs of peritoneal irritation suggestive of small intestinal necrosis. Emergency surgery was performed and the necrotic small intestine was resected. Pathological examination revealed small bowel necrosis due to multifocal HS embolism. The postoperative course was uneventful. The patient was unsuccessfully treated with chemotherapy for HS and died 122 days postoperatively.

**Conclusions:**

HS can cause massive enteric necrosis due to tumor embolism. Clinicians should be aware of this rare presentation of HS.

## Background

Histiocytic sarcoma (HS) is a rare hematological malignancy characterized by morphological and immunophenotypic evidence of histiocytic differentiation [1]. It has an incidence of 0.17 per million, representing less than 1% of all blood lymphoid tumors [2, 3]. HS can affect both lymph nodes and extranodal sites, including the gastrointestinal tract [4], spleen [5], lungs [6], skin [7, 8], and soft tissue [9]. HS affects a wide patient age range, from young to older individuals, with a mean age of onset of 63 years and a slight male predominance [2]. Some studies have reported that patients with localized HS have a good prognosis after curative resection [6, 8, 10]. However, the prognosis of patients with HS is generally poor despite surgical treatment and chemotherapy, with an average overall survival of approximately 6–7.5 months [2]. Herein, we report a case of HS that caused small bowel necrosis due to tumor embolism.

## Case presentation

**Fig. 1:**
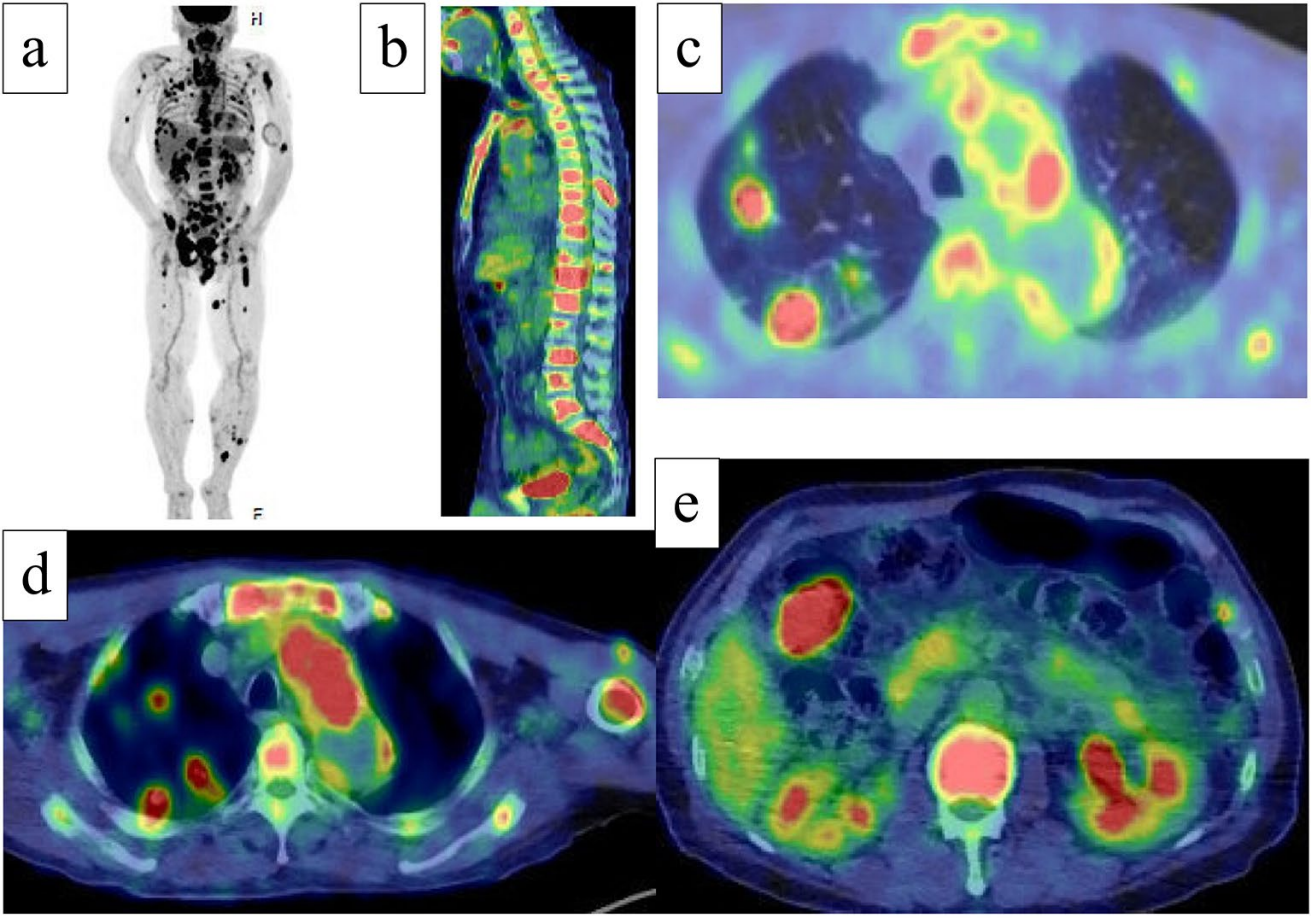
Positron emission tomography-computed tomography showing abnormal accumulation in various organs, such as lung, mediastinum, bone, soft tissue, and intestinal tract (**a** coronal view; **b** sagittal view; **c**–**e** axial view)

**Fig. 2:**
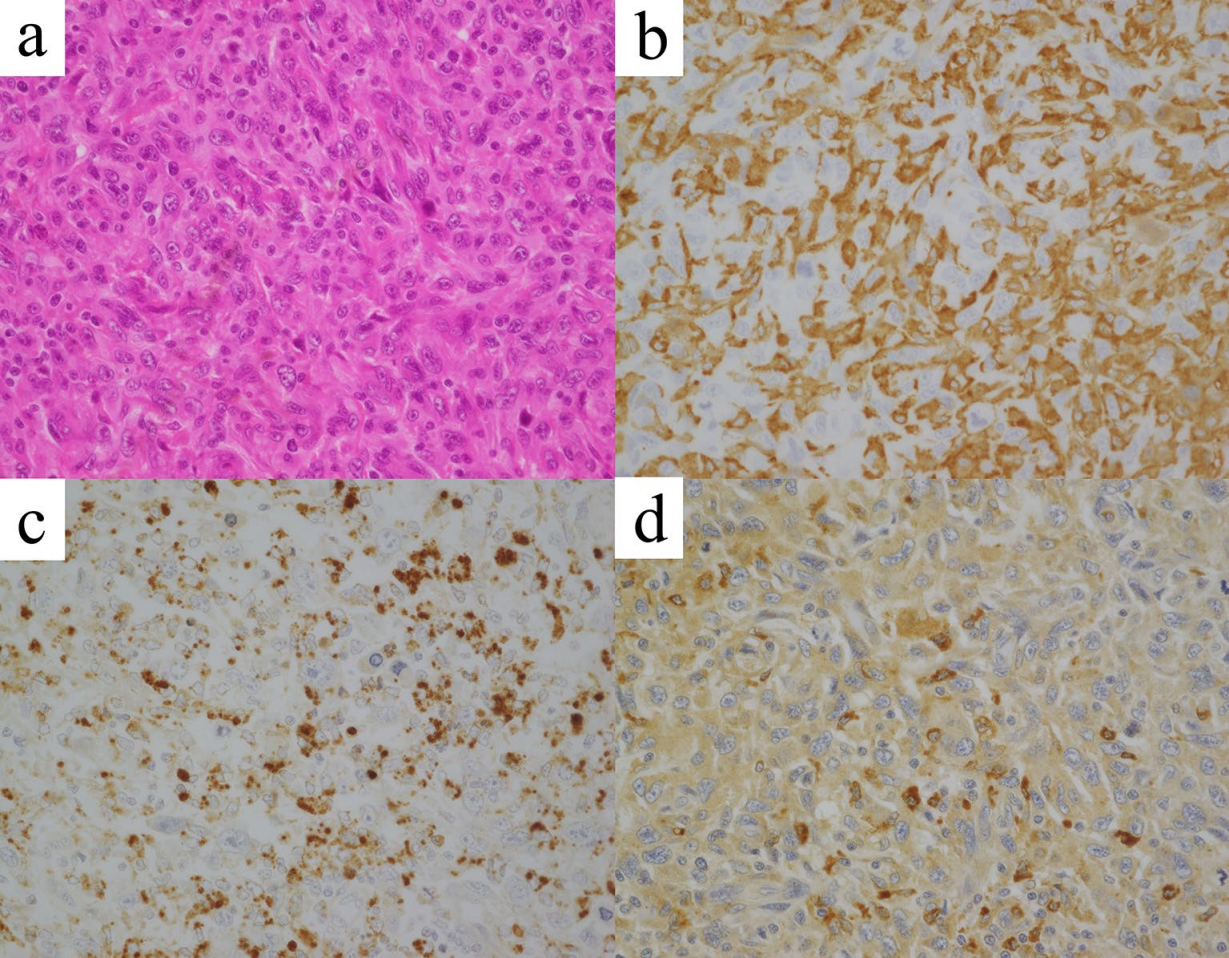
Pathological findings of a subcutaneous nodule on the left lower leg. The hematoxylin–eosin staining shows spindle-shaped tumor cell proliferation (**a**). On immunohistochemistry, the tumor cells were positive for cluster of differentiation 163 (**b**), cluster of differentiation 68 (**c**), and lysozyme (**d**) (× 400)

A 64-year-old man was referred to our hospital for evaluation of early transverse colon cancer. His medical history included hypertension, gout, prostate enlargement, and a previously untreated hepatitis B virus infection. The family history was unremarkable. Initial investigations revealed mild anemia (hemoglobin 11.9 g/dL, leukocyte counts 6100/mL, platelet count 35 × 10^9^/L), mildly elevated C-reactive protein (4.4 mg/dL), and normal tumor markers (carcinoembryonic antigen 1.2 ng/mL, carbohydrate antigen 19–9 6.7 U/mL). The peripheral blood smear did not reveal any abnormalities. The results of the rapid plasma reagin card, *Treponema pallidum* hemagglutination, and fluorescent treponemal antibody absorption tests were positive. Computed tomography (CT) revealed multiple nodules in the lungs, bone, mediastinum, and subcutaneous tissue, along with thickening of the aortic arch wall and left subclavian artery occlusion. Multiple metastases from colon cancer were unlikely because this was early-stage. Proteinase 3 anti-neutrophil cytoplasmic antibody test results were positive. Antinuclear, anti-double-stranded DNA, and anticardiolipin antibodies and rheumatoid factor were negative, and the soluble interleukin-2 receptor and serum immunoglobulin 4 levels were within normal limits. Systemic vasculitis due to syphilis was suspected, and antibacterial treatment with ceftriaxone was administered for three weeks at the Department of Infectious Diseases. A lung biopsy revealed no evidence of vasculitis, and a renal biopsy revealed focal and segmental lesions with no evidence of anti-neutrophil cytoplasmic antibody-associated glomerulonephritis. One week after the end of antibiotic treatment for syphilis, positron emission tomography (PET)-CT showed multiple abnormal accumulations throughout the body (Fig. 1). A biopsy of a subcutaneous nodule on the left lower leg, which was incidentally detected on PET-CT, revealed atypical histiocytic proliferation (Fig. 2). Bone marrow aspiration revealed a hypercellular bone marrow with atypical histiocytic proliferation.

**Fig. 3:**
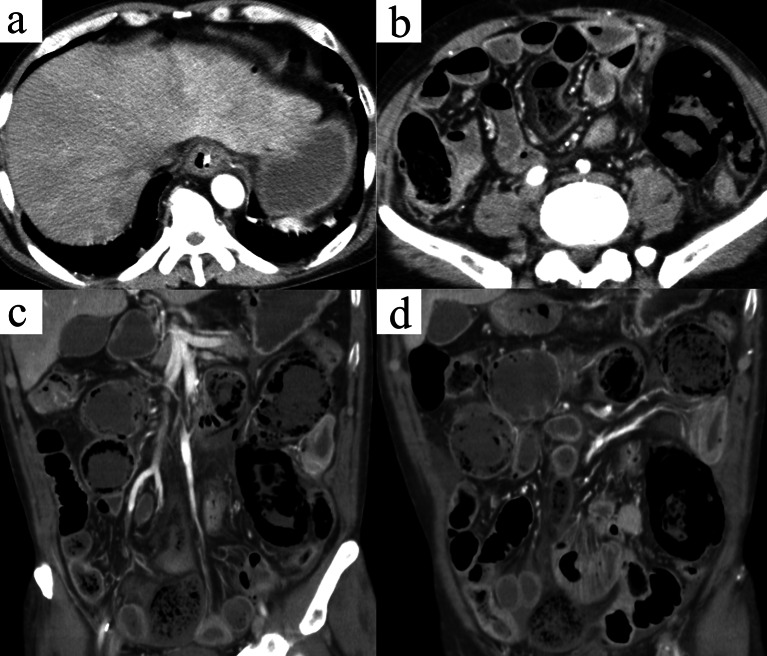
Abdominal contrast-enhanced computed tomography shows free air and ascites in the abdominal cavity (**a**, axial view), emphysematous changes, intestinal dilation, and poorly contrasted areas in part of the small intestine (**b**, axial view; **c** and **d**, coronal view)

**Fig. 4:**
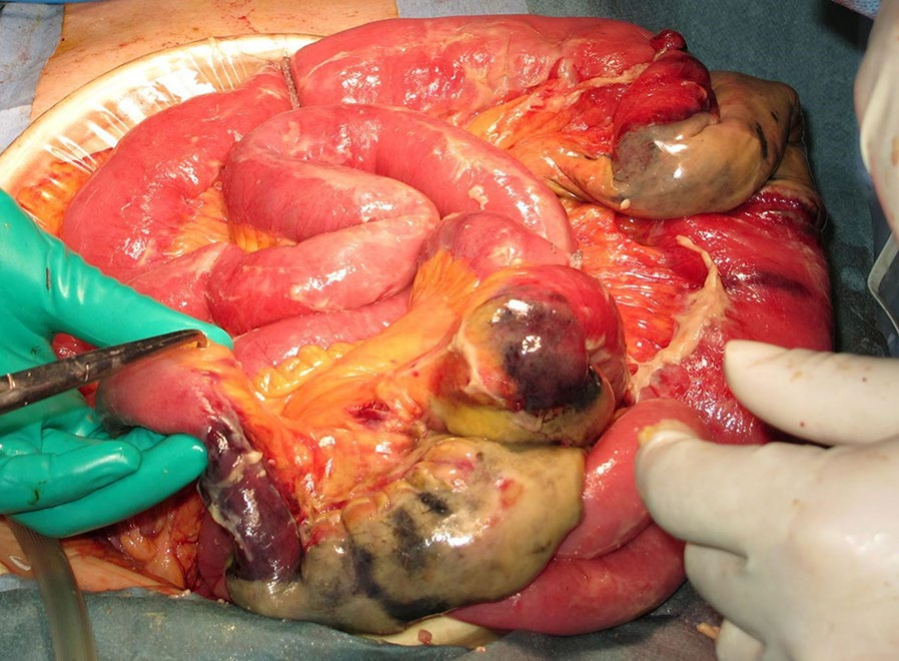
Intraoperative findings. The small intestine is dilated with segmental necrosis, but perforation is not evident

Approximately one month after antibiotic treatment, the patient presented to the emergency department with sudden severe lower abdominal pain and vomiting. On arrival at the emergency room, the patient was conscious, with a blood pressure of 70/55 mmHg, pulse rate of 96 beats/min, and respiratory rate of 48 breaths/min. Physical examination revealed peritoneal irritation and muscular defense. Blood tests showed levels of hemoglobin, 9.2 mg/dL; leukocyte count, 5300/L; C-reactive protein, 22.5 mg/dL; albumin, 1.2 mg/dL; blood urea nitrogen, 61 mg/dL; creatinine, 2.63 mg/dL; and d-dimer, 10.3 µg/mL. CT revealed free air and ascites in the abdominal cavity, a poorly contrasted area in a part of the small intestine, and intramural emphysema (Fig. 3). These findings suggested acute peritonitis associated with small intestinal necrosis and perforation. Emergency laparotomy revealed a moderate amount of turbid ascites with malodor and a dilated small intestine with three dark-red segmental lesions 25–70 cm from the ligament of Treitz, 175–180 cm from the ligament of Treitz, and 12–45 cm from the terminal ileum (Fig. 4). The mesenteric arteries feeding these dark-red necrotic areas were cord-like without pulsation and were suspected to have been embolized by a thrombus or tumor. No apparent intestinal perforations were observed. Three sections of the small intestine were partially resected and functional end-to-end anastomoses were performed at each site.

**Fig. 5:**
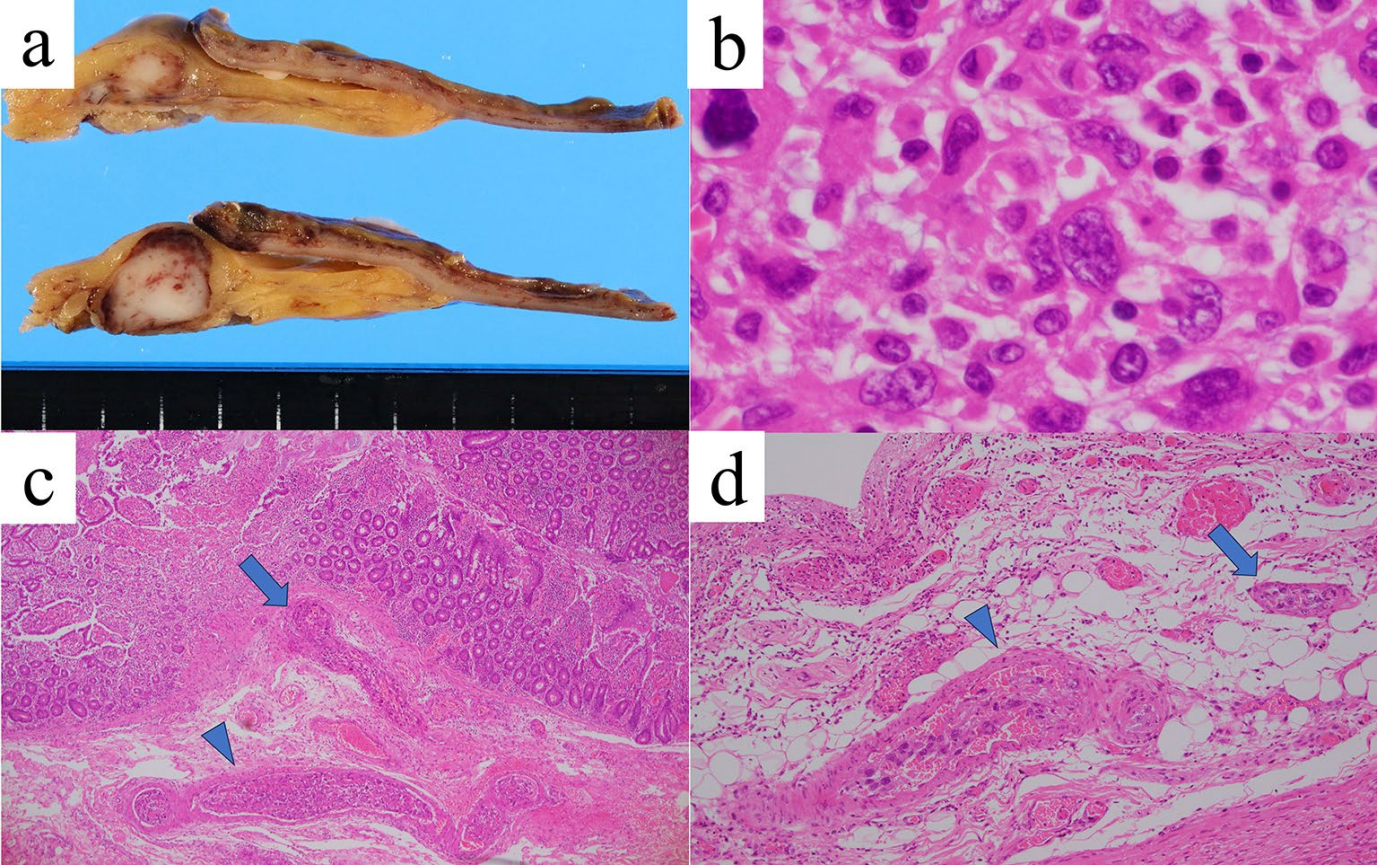
Pathological findings. Macroscopically, a white nodule one cm in size is observed in the small intestinal mesentery (**a**). Hematoxylin–eosin staining shows spindle-shaped tumor cells that grew diffusely and formed nodular lesions with unclear boundaries (**b** × 1000). A thrombus embolism with tumor invasion is observed in the arteries (arrowhead) and veins (arrow) (**c** × 40; d, × 100)

**Fig. 6:**
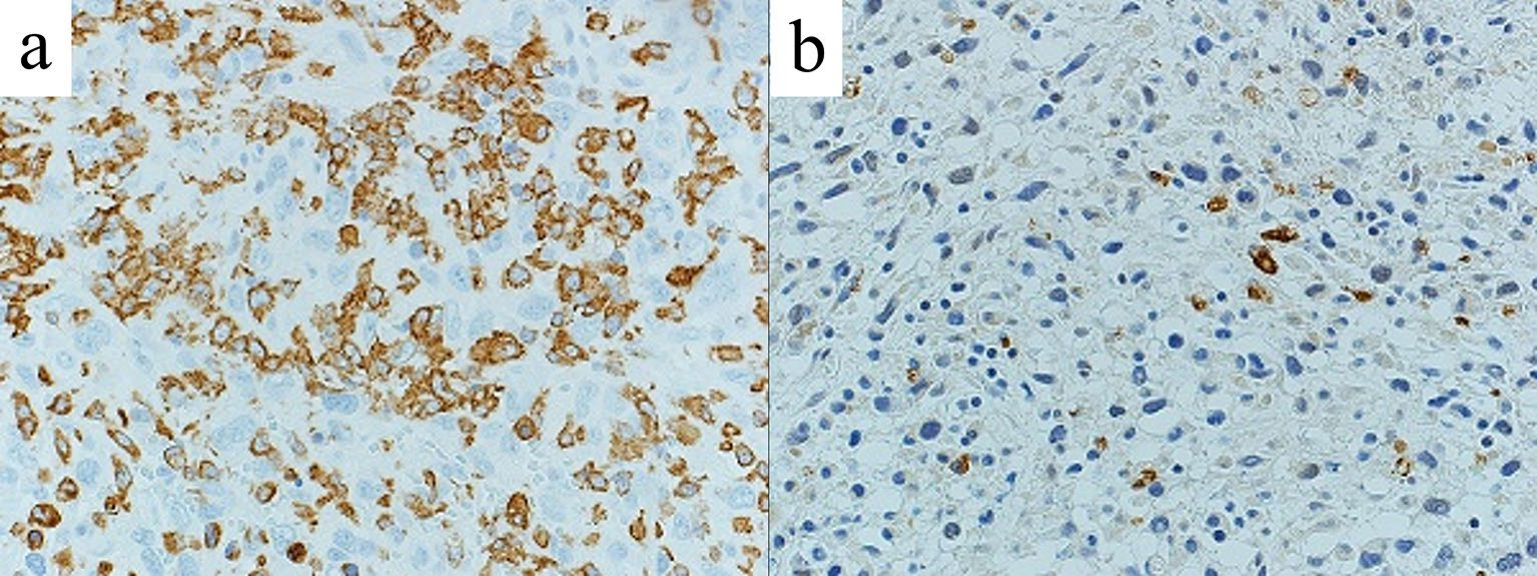
Immunohistochemistry. The tumor cells were positive for cluster of differentiation 163 (**a**) and focally positive for lysozyme (**b**) (× 400)

The resected small intestine exhibited extensive ischemic changes, including regional necrosis. Microscopically, spindle-shaped tumor cells with swollen nuclei increased diffusely and partly in a nodular fashion in the intestinal walls and mesentery (Fig. 5a, b). Tumor cells also massively permeated arteries and veins of various sizes within the intestinal walls, with or without thrombi (Fig. 5c, d). Due to these vascular lesions, the small intestine shows multifocal ischemic necrosis and ulcers, some of which involve the entire thickness of the intestine. Immunohistochemically, the tumor cells were positive for cluster of differentiation (CD) 163 (Fig. 6a) and epithelial membrane antigen, focally positive for lysozyme (Fig. 6b) and CD138, and negative for myeloperoxidase, CD1a, CD21, HHF-35, S-100 protein, and anti-cytokeratin monoclonal antibodies. These results are consistent with the involvement of HSs.

The postoperative course was uneventful and there were no major complications. The patient was transferred to the Department of Hematology on the 9th postoperative day. Cyclophosphamide, doxorubicin, and vincristine were administered as chemotherapy for HS, followed by ifosfamide, carboplatin, and etoposide therapy; however, none of the treatments elicited a response, and the patient progressed to liver and respiratory failure and died of HS 122 days after surgery.

## Discussion

Here, we report a case of HS with an unknown primary lesion and a unique clinical course. A literature search revealed no previous reports of HS with gastrointestinal ischemia caused by a tumor embolism. Tumor embolism is common in lung cancer with invasion into the pulmonary veins or the left atrium because tumor fragments resulting from direct invasion of the tumor into the bloodstream can cause occlusion [11–13]. Systemic HS in this case suggests that tumor fragments invaded through vulnerable blood vessels. The most frequent clinical presentation is acute cerebral stroke, followed by acute myocardial infarction and acute lower limb ischemia [12]. Ischemic enteritis due to tumor embolism is rare but has been reported in cases of lung [11] and rectal [14] cancer.

The symptoms of HS, when present, are nonspecific, including fever, fatigue, night sweats, and weight loss similar to other blood lymphoid tumors. Some previous reports revealed that extranodal multisystemic HS with gastrointestinal lesions might cause intestinal obstruction [15] and perforation [16]. The patient in this case exhibited few symptoms until he was rushed to the emergency department. No findings suggesting tumor embolism were observed on the preoperative contrast-enhanced CT though re-evaluating even after surgery.

Definitive diagnosis of HS was delayed because syphilis was a comorbidity in this case. Multiple nodules were initially identified as systemic manifestations of syphilis based on the laboratory findings, and antibiotics were administered as diagnostic treatment because no other suspicious diseases were evident at the initial treatment. Histological examination is beneficial in differentiating HS from syphilis, so we should have performed multiple pathological examinations of subcutaneous nodules earlier until we confirmed a definitive diagnosis. During laparotomy, necrosis and normal small intestine were distinguished by color tone and arterial pulsation. ICG fluorescence imaging was not used because it is limited to use overtime at our hospital and the boundaries were clear. Resection of transverse colon cancer was not performed because multiple systemic nodules were suspected of malignant neoplasms intraoperatively, which defined the prognosis, but not transverse colon cancer.

When diagnosing HS, it is important to differentiate it from other hematopoietic tumors by immunostaining, following the World Health Organization classification [1]. Immunostaining showed that histiocytic markers, such as CD68, CD163, lysozymes, and S-100 protein, were generally positive, whereas dendritic cell markers, such as CD11c, CD21, and CD35, were negative [3, 9, 17], which is consistent with the findings of our case.

The differential diagnoses include undifferentiated sarcoma with reactive histiocytic proliferation and intravascular lymphoma. In this case, the histiocytes had cytologically malignant nuclei distinct from the bland and distinctive round nuclei, with a fine chromatin pattern found in most reactive histiocytic proliferations [3]. Moreover, this tumor predominantly manifested as an extravascular mass and tumor embolism. This differs from intravascular lymphoma, which predominantly accumulates and proliferates within blood vessels, and rarely forms an extravascular mass [18].

Chemotherapy was initiated for three reasons: First, the surgical intervention was performed immediately. Second, necrosis was localized within a part of the small intestine, and short bowel syndrome was avoided. Finally, the patient did not have ischemic necrosis in other organs due to arterial tumor embolism, cerebral stroke, or cardiac infarction.

Evidence regarding chemotherapy regimens is limited. A few cases of a good response to cyclophosphamide, doxorubicin, and vincristine therapy have been reported [19, 20]. However, the patient showed no obvious response to the chemotherapy. An accumulation of cases of HS and further studies on its treatment are required.

## Conclusion

HS can manifest as a tumor embolism with subsequent massive enteric necrosis. Clinicians should be aware of such rare manifestations of HS.

## Data Availability

Not applicable.

## Abbreviations

HS: Histiocytic sarcoma
CT: Computed tomography
PET: Positron emission tomography
CD: Cluster of differentiation
